# Experiences of participation in bereavement groups from significant others’ perspectives; a qualitative study

**DOI:** 10.1186/s12904-020-00632-y

**Published:** 2020-08-16

**Authors:** Ulla Näppä, Kerstin Björkman-Randström

**Affiliations:** Department of Nursing, Mid Sweden University, S-831 25 Östersund, Sweden

**Keywords:** Bereavement groups, Death, Grief, Palliative care, Qualitative research, Significant others

## Abstract

**Background:**

When death ends a life, the impact of caring for person who suffered a period of illness or disease continues for significant others who are left to grieve. They should be offered support to avoid complicated grief. This can be provided in different ways and individually or in groups. This study aims to describe significant others’ experiences of participation in bereavement groups.

**Methods:**

Ten bereavement groups that each met five times offered support for the significant others of deceased loved ones who had been cared for by a palliative-care team. After the five meetings, the grieving members (*n* = 46) completed written comments about the role of the groups; they also commented one year after participating (*n* = 39). Comments were analyzed with qualitative content analysis with a directed approach using the theory of a good death according to the 6S’s: self-image, self-determination, social relationships, symptom control, synthesis and summation, and surrender.

**Results:**

Bereavement groups were found to be a source for alleviating grief for some significant others, but not all experienced relief. Moreover, grief was found to persist during participation. Another finding involved the impact of the role of the palliative home-care team on bereavement support. To evaluate the experience of participating in a bereavement group, the use the 6S’s as a model was a strength of the analysis. Bereavement groups could enhance the self and offer relief from grief. Participation was described as social relationships that offered a sense of coherence and understanding in grief. The effects of participation were more meaningful close to the loss and could lose efficacy over time. Bereavement support provided before a loved one’s death was seen as valuable.

**Conclusion:**

Overall, the bereavement groups eased the grief of significant others close to the death of their loved one. However, moving forward, several of the significant others were not sure that their participation eased their grief. To identify persons who may remain in a state of complicated grief, a routine of planned contacts with the bereaved should begin before death and be followed up later than six months after the death of a loved one.

## Background

When a person dies, the impact of caring for the person who had suffered a period of illness or disease continues for significant others left to grieve. They enter a period of bereavement and grief. For other bereaved it continues as anticipatory grief as bereavement began before the death of their loved one.

[1]. According to Sweden’s national clinical cancer-care guidelines, all significant others should be offered bereavement support regardless of where or how their loved one died. When the patient has links with a palliative-care team, upcoming bereavement support is routinely offered. The team prepares the significant others for bereavement in natural and careful ways [2]. However, how significant others experience such support requires further research to gain knowledge about how to form effective bereavement groups in health care.

In this report, according to Parkes [3] and Shear, Reynolds, Simon and Zisook [4] the concepts bereavement, significant others, and deceased are used. Bereavement is a state of sadness, grief and mourning that follows the loss of a loved one. In the context of this report, the bereaved is the significant other who is designated the person closest to the deceased, i.e. the person or persons the nurses had met during the loved one’s period of illness or a person suggested by the significant others. He or she has suffered the loss of the deceased, and they need not to be formally related.

### Grief

When the deceased has died following to a chronic illness, the bereaved have time to anticipate and prepare for the death. Some significant others begin their experience of bereavement before death as anticipatory grief, which is a normal response to the anticipation of loss. Significant others start processing the forthcoming loss before the actual death, and this is important for the grieving process [5, 6]. Anticipatory grief can cause feelings of being trapped in the caregiver role, when the death is imminent [7]. It can also place significant others in situations where, cognitively, they recognize the severity of the disease but, on another level, they hold onto the belief that death is avoidable [8]. However, chronic illness may also have the opposite effect; significant others fail to anticipate death because of the long-term nature of the illness, and they adapt to the situation as being normal [9].

Grief is not a uniform concept; it differs depending on an individual’s personality, cultural and spiritual beliefs, coping skills, socioeconomic status, and availability of bereavement-support systems. Although most people experience normal grief and cope well. Research has shown that, after 6 months, it can be distinguished from complicated grief [6, 10]. Complicated grief is characterized by continuing severe distress and dysfunctional thoughts, feelings or behaviours related to the loss. A feeling of yearning persists and includes a strong desire to be with the deceased, along with an inability or refusal to accept his or her death, preoccupation with thoughts about the deceased, and a habit of keeping reminders close. Complicating factors are rumination about the death and unrelenting bitterness or anger. Emotional reactivity to or avoidance of reminders and social estrangement are common, as are suicidal thoughts, since life seems empty and meaningless, and joy is impossible [5, 10]. It may be difficult to distinguish grief from depression because thoughts of death and feelings of guilt can cause both conditions [6]. Complicated grief and bereavement have also been associated with increased physical morbidity such as cardiovascular events, pulmonary embolism, and acute coronary syndromes [5].

### A good death

The aim of bereavement support is to ease the process of grieving and strengthen the ability of the significant others to cope [7]. In Sweden, the 6S model for care planning, documenting, and evaluating the quality of death originating from person-centred care, was developed aiming to connect to a nursing context [8, 9]. The source of the model is the World Health Organization’s definition of palliative care [11] and Weisman and Kastenbaum’s [12] questions for retrospectively determining whether the deceased had an appropriate death. The central point of the criteria is the personal focus. Weisman and Kastenbaum suggested that an appropriate death may be attained by preserving a person’s self-esteem and respecting his or her individuality for as long as possible; then, he or she may face death with the same dignity as life [12].

### The 6S model

The 6S model comprises six keywords (see Table 1) and was developed as a nursing model of care from the perspective of person-centred care [9]. In person-centred care, seeking information about who the patient is and his or her preferences for life or for a good death are of great significance.

**Table 1:** Content of the 6S Model [8, 9]

Self-image	The individual person’s self-description – who is the person and what is of central importance in his or her life?
Self-determination	The individual person’s wishes and needs to be a co-actor in his or her life. How dependent is the person, and is there a way to support him or her to achieve autonomy?
Social relationships	Ways to support the individual person to maintain important social relationships in the way he or she wishes.
Symptom control	The individual person’s experiences of physical and bodily needs. This includes experiences of unpleasant physical changes that need to be alleviated.
Synthesis and summation	Seeing the individual person’s spiritual and existential needs in retrospect and creating continuity and meaning in the death of a significant other.
Surrender	Seeing the individual person’s spiritual and existential needs moving forward and creating meaning and acceptance.

Seeking information about the significant other’s preferences for the care of a terminally ill loved one offers the same measure of dignity [9, 13].

### Bereavement support

Literature reviews show that bereavement support is offered in different ways, for example, as individual sessions, family therapy, follow-ups to bereavement-group programmes, voluntary bereavement groups, breathing and stretching sessions, relaxation and meditation, telephone calls, chat groups or invitations to memorial ceremonies. Counsellors involved in bereavement support can represent many different professions; they may be registered nurses, clinicians, clinical psychologists, social workers, research assistants, trained professional counsellors, volunteer bereavement counsellors, trained staff and family caregivers, formal caregivers or hospice staff [14–16]. In addition, members of the clergy and deacons are represented among grief counsellors [17]. A review by Currier, Neimeyer and Berman [18] showed that universal bereavement support, targeting anyone in bereavement, may ease grief in a post-participating phase but not in follow-up. When bereavement support was provided to a targeted population at risk of experiencing symptoms of distress related to the loss of a significant other or violent death, the groups had a significant overall effect (ibid.). Receptivity towards bereavement support depends on a number of factors at the individual, societal and geographic levels. Bereaved persons described support based on shared trauma with other bereaved persons in similar situations. However, not all bereaved persons want to share their loss with family-support professionals [19], and others do not others do not experience the need for bereavement support from health care professionals, as they feel they have adequate support from family and friends [17, 20]. Yet significant others can struggle following the death of a loved one if they have a desire to talk to somebody and do not know where to get help [21]. Aoun, Breen, White, Rumbold and Kellehear [22] found that informal support from family, friends or funeral providers was perceived as very helpful (92–88.3%: *n* = 678). The same study found that bereavement-support groups were perceived as helpful by 58.8% of participants.

Specific interventions in bereavement support that investigate, for example, children’s or youths’ loss of a parent or parents’ loss of a child have been described [14, 16]. However, research found that, in rural areas, providing support was difficult and sometimes impossible to tailor according to the kind of loss experienced [17, 23, 24]. Furthermore, some significant others may not benefit from participating in blended bereavement groups [23]. These factors require further research about bereavement support for persons in general where predefined groups are not possible.

## Aim

The aim of this study is to describe significant others’ experiences of participation in bereavement groups.

## Methods

### Setting

The study setting was a county in Northern Sweden with about 130,000 inhabitants in an area of 49,935 km^2^ (< 1% of the total Swedish population and 2.7 inhabitants per km^2^). The county is characterized by mountains, rural areas, and an urban area close to the only city in the middle of the region. One palliative home-care team comprising registered nurses and physicians specializing in palliative care was based at the hospital that served the whole county. This team, in cooperation with the district nurses, made it possible for persons to die at home. In almost all cases, significant others were spending time with the patients in their final days of life. This team treats approximately 150 persons annually. The distances from the hospital, where the team is located to the farthest patient could be 250 km one way. When a person who was cared for by the palliative home-care team died, the nurses contacted the significant others connected to the deceased, i.e. the person or persons they had met during the loved one’s illness or a person suggested by the significant others. They informed these people that an invitation to a bereavement group held at the hospital would arrive within three to 6 months after the death of their loved one. Accordingly, within that time frame, the social workers and clergy members or deacons working at the hospital extended an invitation to the bereavement group by telephone. About one-third of the significant others invited accepted the invitation, resulting in groups generally comprising 7–12 participants and 2–3 counsellors.

The bereavement groups followed the same rationale recommended by the Swedish Church, with an underlying theory of potentially positive results through the verbalization and re-exposure to grief in a safe group context. Each of the five meetings had a predefined theme: 1) a presentation of the methodology and an introduction to the group’s members; 2) a focus on the period of the loved one’s illness before death; 3) a focus on the time at death; 4) a focus on the period following the death and the funeral; and 5) a depiction of a metaphorical picture of the deceased and the significant other’s life together [25]. The groups met for2 h once a week for 5 weeks and included afternoon tea. Counsellors for the groups included a nurse from the palliative home-care team, a social worker, and a clergy member or deacon.

Counsellors’ salaries were paid entirely by tax money, with no charitable or other contributions. All had several years of clinical experience meeting with and counselling the bereaved significant others of palliative-care patients. Social workers and clergy members or deacons have formal education in counselling, while registered nurses broach the subject during their basic training. The role of the counsellors was to listen rather than to lecture and to ensure that all of the participants had opportunities to take part in the discussions. They steered the discussions towards the expression of thoughts and emotions. In a small number of meetings, the nurse serving as a support professional had been caring for the deceased. A very small number of significant others might have met the social worker or clergy member or deacon during the course of their loved one’s illness. At the same time, the Swedish Church also provided bereavement groups with the same concept to bereaved persons, independent of the cause of death. Leaders for these groups were paid by the Swedish Church, and meetings were held in different parishes, some geographically closer to the bereaved individuals than others.

### Data collection

This study is part of a larger project that aimed to analyze quantitatively the effects of bereavement groups on grief, anxiety and depression [17]. A total of 124 bereaved significant others invited to 10 bereavement groups completed questionnaires that utilized the Texas Revised Inventory of Grief (TRIG) [26], from which 13 questions were used in the study; the Hospital and Anxiety Depression Scale (HADS) [27, 28], with 14 questions; background questions; and open questions.

One of the open questions gave the significant others participating in the bereavement groups the opportunity to offer their opinions about the role of the groups in their bereavement. The wording for the question was as follows: “If you participated in the bereavement group, what role has it had in your processing of grief?” Most participants wrote more than five sentences; only a few answered in only one sentence. The total data set resulted in a word count of 3652 words, divided into 158 meaning units. Questionnaires with this open question were completed 5 weeks post-participation and 1 year later as a follow-up. The open question was not qualitatively analyzed earlier. The post-participation (*n* = 46) answers and follow-up (*n* = 39) answers are analyzed in this study. For sociodemographic data related to the significant others, see Table 2.

**Table 2:** Sociodemographic data of significant others

Gender	Female, *n* = 33	Male, *n* = 13
Age		
Median	64,5 y	66 y
Range	43 y – 80 y	49 y – 75 y
Relationship – the deceased was a:		
Spouse	24	11
Parent	6	2
Child	1	
Other	2	
Distance to hospital where bereavement groups were held		
< 60 km	19	10
60–109 km	2	2
> 109 km	5	1
Highest education		
Primary school	8	
Secondary school	6	3
Vocational training	7	9
University	10	1
Working status at time of post-participating questionnaire		
Working	9	6
Working part time + sick leave	1	
Sick leave 100%	5	
Retired	17	7

### Data analysis

Reading the written comments made the researchers consider how to analyze them. A qualitative content analysis with a directed approach [29] was performed on the answers to the open question “If you participated in the bereavement group, what role has it had in your processing of grief?” using the predetermined structure of the 6S keywords. The 6S seemed to be a relevant tool, in this concept evaluating the bereaved persons’ feelings of a good death [8, 9]. The analysis included several steps. First, all open answers were read several times to acquire a sense of the message. Second, the text was divided into meaning units consisting of a sentence, several sentences or a paragraph with similar meanings related to the aim of the study. Third, these meaning units were compared in order to identify similarities and differences, and then categorized according to their proximity to each of the 6S’s [8, 9]. The two authors (UN, KBR) performed the analysis and discussed minor disagreements in a critical dialogue until consensus was achieved. Quotations are presented in the results section with the words used by the bereaved persons in italics. All persons are given pseudonyms.

## Results

The findings emanated from the open-ended answers to the question “If you participated in the bereavement group, what role has it had in your processing of grief?” They show that the bereavement groups gave significant others opportunities to share their inner feelings. Another finding involved the impact of the role of the palliative home-care team on bereavement; see Table 3.

**Table 3:** Result of the study

**6S**	**Categories**
Self-image	Feeling of being seen
	Understanding own identity
	Moving on from grief
Self-determination	Being co-actor
	Regaining harmony with self
	Comparing with others
Social relationships	Insights about others
	Composition of groups
	Needs of further support
Symptom control	Needs of medication
Synthesis and summation	Creating meaning
	Feeling of grief
Surrender	Passing through phases
	Life goes on
**A finding outside of the 6S’s**	**Category**
The role of the palliative-care team	Being a lifeline

### Self-image

In regard to self-image, significant others described their experiences of participating in bereavement groups as a positive form of support. The bereavement group contributed to a feeling of being seen by others and provided increased understanding of their own identities. The group was found to be valuable in a challenging situation. Mr. A, whose age was between 61 and 70 years, commented 5 weeks post-participation: *I [now] better understand that those who do not have someone in their own household to share their grief with have much more difficulty. I can more easily accept that I was present at the very moment of death.*

Other significant others expressed receiving no or minimal support in regard to cognitive and emotional aspects. They seemed unable to move on from their grief 5 weeks post-participation: *I still have a hard time and I cry a lot, but I guess that’s the way I am* (Mrs. B, age 61–70 years). Over time, the follow-up answers illustrated that the groups gave the participants a feeling of commonality but that grief persisted. Mrs. C, age 61–70, wrote: *However, grief goes up and down and it needs to do so. A big thank you to everyone who attended the meetings and for the support we received.*

### Self-determination

Significant others described their experiences of participating in bereavement groups as a forum for strengthening their self-determination. The bereavement group supported them in regaining a sense of harmony within themselves by being co-actors in the group. Participation was described this way 5 weeks post-participation: *It meant a lot to set words on what happened. Painful memories came up to the surface again, but it was also nice to talk about them [the deceased]. By narrating, we made him “alive” again* (Mrs. E, age 41–50).

Significant others also compared themselves to others in the group. These comparisons were mostly positive, but some did not want to share their grief in this setting. Mr. F, age 61–70, wrote 5 weeks post-participation: *I do not want to discuss my grief with unknown people; I process it by myself and with my relatives.* Thus, he participated only twice. Other significant others commented that they also needed other kinds of support, for example, individual contact with a psychologist. Over time the follow-up comments about the benefits of participation in the bereavement groups were both positive and negative.

### Social relationships

Bereavement groups confirmed social needs and that significant others could depend on each other. They gained insights about other persons’ journeys through the grieving process and those who were in the same stage of grieving as they were. Mr. G, age 51–60, wrote 5 weeks post-participation*: [I] found out that we are several in the same situation as me. You are not alone.* Significant others also sensed positive support from the counsellors. The continuity and composition of the groups were important, and participants offered advice about the arrangement 5 weeks post-participation including: *Good arrangement. Possibly smaller groups – maximum of five significant others. Missed “my” nurse from the palliative-care team – the feeling of belonging that was built up during the illness* (Mrs. H, age 51–60). Over time in the follow-up, the significant others wrote about the positive feelings of sharing experiences, but in addition, a need for further support was declared. They asked for support from either some kind of psychologist or from the palliative home-care team. Mrs. I, age 51–60, illustrated this: *I do not know how only the bereavement group has influenced my grief. However, together with appointments with my psychologist, it has been very good for me. I do not close up anything. I hope that these bereavement groups will continue and expand to be additional. We are many who need support and help. You have all done fantastic work!*

### Symptom control

Only one significant other mentioned a physical symptom in the post-participation questionnaires 5 weeks after the bereavement groups. Mrs. D, age 71–80, wrote: *Now I feel calmer and can sleep without sleeping pills,* thereby, expressing positive benefits from participating in the bereavement group. Over time in the follow-up, significant others related that the groups had been premature in relation to their declining physical and/or mental health. Starting with adequate medication was helpful; Mrs. J, age 71–80, wrote: *None [effect of the bereavement group]; I think it came a little too close to the death. I got depression in the early summer. I now medicate with Citalopram 20 mg and feel healthy and strong again.*

### Synthesis and summation

The group provided support for the significant others’ retrospective existential needs and created meaning in the present. Mrs. K, age 61–70, wrote 5 weeks post-participation*: It [the bereavement-group] had a great impact, where each one could tell and cry about the trauma that everyone had experienced. We became like a big family, who felt the same grieve after the loss of our loved one died. I felt confidence from the leaders who were there.*

The reflections that arose in the bereavement groups in regard to being in a phase of existential loneliness were experienced as support, but the feeling of grieve was still present in everyday life 5 weeks post-participation. *It was of great value to me, but the grief is still just as big* (Mrs. L, age 61–70).

Over time, the answers showed that some of the significant others did not get on with their lives but rather continued to feel grieve. Mr. F, age 61–70 years, wrote in the follow-up: *Not much [benefit from the bereavement-group] more than that, I was told about other peoples’ grief. However, every grief and experience is unique to that person.*

### Surrender

In regard to the need for support, the answers revealed that the death of their loved one was always with them, but that the bereavement group offered great support. The bereavement group’s structure of five meetings over time was designed for passing through the different phases of grieving up to the death, at the time of death, and the period following the death and the funeral 5 weeks post-participation. *The bereavement groups fulfil a great function. I began healing and gained insight on how others experienced their grief* (Mrs. M, age 61–70).

Participation in bereavement groups gave significant others strategies to help them realize that life goes on and grief reaches closure, although it is always present. Mrs. N, age 71–80, wrote in the follow-up: *The grief must take “its time”*.

Significant others expressed at both occasions that the counsellors helped the participants express their inner feelings about grief in the present. They also offered counsellors advice about the composition of the groups; they suggested that widows and widowers should have been in one group and younger bereaved persons should have had their own group. An additional number of meetings was also suggested to ease the bereavement process.

### The role of the palliative-care team

There appeared to be two different experiences of support in bereavement – support from the bereavement group and support from the palliative home-care team. The support from the palliative home-care team was a finding outside of the 6S’s.

The bereavement groups were considered very valuable for providing a context for grief. However, the answers also indicated the high value placed on the earlier close contact with the palliative home-care team. The team had provided a sense of meaning to the significant others related to difficult situations in life and appeared to serve as a kind of lifeline for grief in both the 5 weeks post-participation questionnaires and in the follow-up. *Thank you for being there. [...] What would I have done if the palliative-care team had not been there; I cannot think of that. You do an incredible job. You are the relatives’ lifeline and comfort. Thank you for being there* (Mrs. K, age 61–70).

## Discussion

Care planning for grieving significant others is as important as the care of the dying person. To be strong and to succeed in going on with one’s life requires the individual to affirm his or her self-image, self-determination, social relationships, symptom control, synthesis and summation, and surrender. The 6S model, therefore, was found to be a well-functioning tool for analyzing grief among significant others.

The findings of the present study indicate that the bereavement groups, in some sense, contributed to strengthening the significant others’ self-images and gave them a better understanding of themselves. The groups gave participants opportunities to process the story of the death and to make sense of it and of their upcoming lives without their loved one. This aligns with international grief therapy as described by Neimeyer [30].

However, not all significant others used the bereavement groups as a catalyst to affirm their self-image.

Whether these significant others had come to terms with the imminent death is unknown. Holland, Futterman, Thompson, Moran, and Gallagher-Thompson [31] suggested that non-acceptance of the upcoming loss when the patient is still alive is a predictor for negative grief experiences later. Identifying those individuals who are likely to benefit from bereavement services should be part of the palliative-care process before the death of their loved one. Other findings have shown, for example, that psycho-educational group interventions for significant others before death can increase competence for caregiving and preparedness for the loved one’s end of life [32]. Nurses and physicians in palliative care should pay close attention to expressions of grief in the early aftermath of loss. However, participation in groups is related to the date of death and can lose its effectiveness in the long term. Findings confirm that, over time, significant others are hesitant about whether the bereavement groups offer any support at all [31, 32]. However, Blackburn, McGrath, and Bulsara [33] found that significant others can struggle with difficulties for longer periods than formal support services are aware of. Follow-up support to some significant others may need to be continued for a longer time. Blackburn et al.’s suggestion is that the community should provide that kind of bereavement support.

To reveal one’s inner feelings in a group can be difficult and may require self-determination. In bereavement groups, significant others had the opportunity to decide whether they wanted to discuss their grief and share their inner feelings. Hefren and Thyer [34] recommended guided bereavement support, which may be helpful for relieving participants’ negative emotions and avoidance behaviours. Guided support can be valuable for identifying grief and enhancing bereavement even in complicated grief.

Feelings of loneliness and isolation are common, and many times, the bereaved struggle with these feelings. Many among the bereaved participants had been half of a couple (Table 2), and the loss of a spouse or partner can be a risk factor for prolonged grief disruption. Participation in bereavement groups can improve relational functioning in the post-treatment phase and well-being at follow-up [18]. Social support is a source of finding a meaning and can ease the risk of prolonged grief [35]. Being a member of a bereavement group is a form of social support. Research shows that being an ageing couple can imply that friendships outside the relationship are lacking. The spouse and family members are the only social network [36]. However, this study’s findings illustrate that the bereavement groups had a positive influence on social relationships close to the death of the deceased. Significant others experienced that participation as providing a sense of coherence and understanding that they could share with others in the same situation in connection to their loved one’s death, as confirmed by Blackburn and Bulsara [19]. Findings from this study show that social relationships benefitted by meeting other persons in the same situation in bereavement groups. Participation can give valuable meaning in life. The loss of a spouse or partner has been shown to be significantly negatively correlated with having meaning in life (−.25, −.29: *n* = 171) and with prolonged grief disorder (.36: *n* = 171) [35].

The findings of Oliver et al. [37] showed that using technology in bereavement can be beneficial. On Facebook, bereaved significant others shared confidential posts in private groups with a social worker as the facilitator. Members’ relationships with the deceased influenced the group’s responses; in Oliver et al.’s study, spouses offered significantly more support to each other. In addition, newly bereaved significant others received posts from significant others who suffered bereavement earlier (ibid). Sharing bereavement through social media can be a way to offer persons living far away from bereavement groups an opportunity to manage to go through the death of a loved one.

Symptom control is also a key term in palliative care for both patients and significant others and is habitually seen as physical needs [5, 11]. However, the findings in this study did not seem to indicate that the symptoms of physical needs were alleviated. Rather, the benefit seemed to be psychological. This aligns with Bergman et al.’s review [14] about bereaved children who showed no physical distress in grief.

Findings from the present study demonstrate that bereavement groups were a source of a feeling of synthesis in the retrospect of the loved ones’ death. However, significant others also gave valuable advice about how to assemble the groups. Not all were comfortable in a blended group or with the time at which the bereavement process was offered. Developing groups can be challenging in rural areas as the number of bereaved is small, and thus, the number of those in need of bereavement groups is even smaller. Findings from rural Australia also confirm this. In rural areas, all significant others were offered the same bereavement support, while, in urban areas, bereavement support could be offered, for example, solely for those who were grieving the loss of a loved one from leukaemia; this distinction was appreciated by participants [23]. The earlier study by Näppä et al. [17] revealed that living a large geographic distance from the groups could be a reason for not participating in bereavement groups. In the Internet age, a way to manage time and distances might be the use of online services for bereavement. This already exists but is, in general, directed to specific groups, for example, bereaved parents after the loss of a child [33, 34] or bereaved children, for example, as through CanTeen [38], a website that has a page for children with parents suffering from cancer.

This study shows that bereavement groups gave significant others the tools to accept the loss they had suffered and recognize that life must go on. Group membership has been found to provide therapeutic benefits. Small, time-limited groups can lead to intimacy and cohesiveness and promote mutual aid and support. Members in the groups benefit from opportunities to give, not just receive, assistance in the form of support, understanding, comfort, and suggestions about how to go on in life. In the long run, this empowers the bereaved and enhances their feelings of self-efficacy and ability to cope in bereavement [36, 39]. However, earlier research has shown that bereavement interventions provide therapeutic outcomes close to the post-intervention time but fail to last in follow-up [18].

The findings from this study also demonstrate that the role of the palliative-care team was unexpected. Significant others experienced the support from the palliative-care team as more valuable than that from the bereavement group. The relationship that developed earlier seemed to be more valuable, yet they participated in the bereavement groups. Other research has confirmed that the close, pre-existing relationships that develop between palliative-care teams and nurses working in home-care and the family members of the patients are highly valuable. The interactions create relationships characterized by mutual sharing of understanding and trust, almost like a family [19, 40]. Aoun, Rumbold, Howting, Bolleter, and Breen [41] found that about half of the significant others who had received support from a palliative-care team (*n* = 298) felt they were given as much help as they needed from the team. Of these, only 10% participated in bereavement groups.

However, support provided by palliative-care teams is strongly related to available resources and opportunities. Caring for bereaved significant others for a long period will take resources away from the primary task of such teams – providing palliative care for living patients. Therefore, it is important to be clear regarding the set-up of work by the team while the care is on-going.

Implementing some kind of program, for example, the one described by Holm et al. [32] with three sessions by health professionals involving significant others in palliative caregiving, can be a way to support the lives of significant others following the death of a loved one.

### Methodological considerations

Using the 6S for analysis may be controversial since it was developed with the goal of having a good death. Our belief is that, if the significant others experienced that their loved ones had had a good death, their own experience of grief could be connected to the 6S’s. Using this model provided a systematic structure to the analysis that was easily repeated. Studying the effectiveness of bereavement support should align with the actual settings to the greatest extent possible [42]. The aim of the bereavement groups studied in this research was to provide a sense of the loved one having had a good death.

Written comments without the possibility of asking follow-up questions are always a one-way type of communication, the interviewer would have had opportunities to further develop the questions and gain more-complete responses. As it was, written comments gave the significant others opportunities to describe their experiences in regard to the influence of bereavement groups and their thoughts about the value of these groups. The time it took for participants to sit down with pen and paper gave them a chance to reflect and then record their thoughts and inner feelings without the stress an interviewer’s presence may have caused them.

## Conclusion

According to the findings viewed through the lens of the 6S model, different kinds of support are needed by those participating in bereavement groups. An advantage of participating in such groups was that they helped for the significant others to capture and share expressions for enhancing their self-image, self-determination and their needs for synthesis and summation. Significant others experienced participating in bereavement groups as a recognition of each other’s grief and moving forward in the process of creating meaning and acceptance in daily life.

Overall, the bereavement groups showed that the grief of significant others close to the death of their loved one could be eased. However, moving forward, several of the significant others were not sure that their participation eased their grief. In addition, whether the groups help to ease grief over the long term is uncertain. Significant others may remain in a state of complicated grief. To identify these persons, a routine of planned contacts with the bereaved should begin before the death of the loved one in a controlled way and be followed up later than 6 months post-death. Bereavement support was also experienced from the palliative-care team who had met the significant others before the death. The care from the palliative home-care team was considered highly valuable. The findings of this study have provided valuable knowledge about the needs of bereavement support for significant others participating in bereavement groups and have implications for health care and social welfare.

## Data Availability

The datasets used and/or analyzed during the current study are available from the corresponding author on reasonable request.

## Abbreviations

HADS: Hospital and anxiety depression scale [27, 28]
TRIG: Texas revised inventory of grief [26]
y: Years
